# A narrative review of peer support barriers and facilitators in kidney care

**DOI:** 10.1111/jorc.12350

**Published:** 2020-10-08

**Authors:** Andrew Trasolini, Eleri Wood, Nicola Thomas

**Affiliations:** ^1^ School of Health and Social Care London South Bank University London United Kingdom of Great Britain and Northern Ireland; ^2^ King's College Hospital NHS Foundation Trust London United Kingdom of Great Britain and Northern Ireland

**Keywords:** barrier, chronic kidney disease, facilitator, peer support

## Abstract

**Introduction:**

Patients with chronic kidney disease often experience emotional/mental challenges and benefit from peer support, as it provides insight/information from others with the same condition. Previous studies show it is effective in improving health outcomes and aids in treatment decisions.

**Literature Review:**

There is low peer support uptake among patients with chronic kidney disease in the United Kingdom and staff do not utilise peer support services fully. Few studies within the United Kingdom have focused on peer support barriers/facilitators, so this narrative review aimed to understand them from staff and patient perspectives.

**Materials and Methods:**

A comprehensive search strategy and inclusion/exclusion criteria were applied with a two‐step process of article selection employed using two reviewers. Thematic analysis was applied.

**Results:**

Five articles were included and six themes emerged. Low referrals and difficulty matching were staff barriers; concern regarding the relationship dynamic and the format/delivery were patient barriers. Promotion of the service aided the uptake from staff, while patients valued inclusivity.

**Discussion:**

Increased promotion of peer support benefits through training/awareness may improve staff referrals and there should be greater exposure nationally. A flexible format is essential to ensure ample opportunity for access.

**Conclusion:**

This review highlights the current literature on peer support barriers/facilitators. Further study is needed to evidence which approaches best overcome staff‐ and patient‐barriers.

## INTRODUCTION

The experience of being diagnosed with chronic kidney disease (CKD) can often be overwhelming as it requires a number of behavioural changes and can be accompanied by both emotional and mental challenges (Morton, Tong, Howard, Snelling, & Webster, 2010). The complexity of the condition and the resulting impact on the individual and family may affect the quality of life and independence (Burnier, Pruijm, Wuerzner, & Santschi, 2015). To provide opportunities for people with CKD to overcome such adversity, in the UK, peer support (PS) programmes are policy‐recommended (Department of Health, 2013), as they can help people with the same chronic condition to gain insight from one another through the sharing of experiences and information (Perry et al., 2005). Additionally, since the National Health Service (NHS) in the United Kingdom faces repeated budgetary and staff shortages, PS is a suitable and low‐cost supplement to standard clinical services on kidney units (Wood, 2014).

PS programmes have been shown to be effective in helping people manage long‐term conditions, such as mental health disorders, cancer and HIV/AIDS. The type and duration of PS can be structured differently depending on the environment and goals of the service but can resemble teaching, mentoring, buddying, one‐to‐one support or group sessions (Hughes, Wood, & Smith, 2009). PS is classified as informal, conducted by a fellow patient, or formal, implemented by a fully trained peer supporter. More specifically, Ghahramani (2015, p. 241) has broken down PS into seven categories: “professional‐led group visits; peer‐led self‐management training; peer coaches; community health workers; support groups; telephone‐based peer support; and web‐ and email‐based programs.”

A person's response to receiving PS is often positive, as recipients can learn coping strategies and ways of managing their condition (Winterbottom, Bekker, Conner, & Mooney, 2012). Supporters who have lived experience of CKD are in an advantageous position to address questions and concerns of newly diagnosed patients in comparison with general healthcare professionals as they have a stronger sense of awareness and relatability (Heisler, 2006). Previous studies on peer mentoring between individuals with CKD have evidenced benefits such as providing a source of information for people and aiding PS recipients in feeling heard and understood given the supporters' authentic experience of the condition (Wood, 2014). It has also shown to improve self‐efficacy and emotional well‐being (Hughes et al., 2009), aid in treatment decisions (Hughes et al., 2009; Winterbottom et al., 2012) and relieve certain fears regarding specific procedures and therapies (Winterbottom et al., 2012). Additionally, trust can be developed among recipients of PS and has shown to decrease health disparities, especially among disadvantaged groups (Perry et al., 2005). Despite claims in the past that peer supporters might provide erroneous information, this can be combatted by ensuring peer supporters are trained on a regular basis and advised against delivering any medical‐related advice (Wood, 2014).

Although PS is generally well‐received by PS recipients, there is low uptake for formal PS among patients with CKD in the United Kingdom (Hughes et al., 2009; Taylor, Gutteridge, & Willis, 2016; Wood, 2014). The problem is not unique to PS in kidney care. Accordingly, National Voices (2015) in the UK put out a nationwide call to utilise both volunteer and community groups to produce more opportunities for PS.

### Aims of the review

To increase overall participation in PS programmes within kidney units across the United Kingdom, it is imperative to first understand the patient and staff perspectives, which either deter or facilitate access to PS opportunities (Taylor et al., 2016). Once identified, these particular aspects can then be addressed, with alternative solutions proposed, if appropriate. A number of studies have focused on barriers and/or facilitators to PS uptake in geographic areas like the United States (Liaghat, 2017) and Canada (Nicholas et al., 2009), but fewer have been cited within the United Kingdom. A narrative review format was therefore chosen so a UK‐specific perspective of current literature could be better understood, with emerging themes presented.

### Review question

This narrative review was conducted to synthesise what is presently understood, surrounding both healthcare staff‐ and patient‐related barriers and facilitators to PS for people with CKD in the United Kingdom, to influence future practice and recommendations within this field. The review question “What are the barriers and facilitators to accessing peer support in kidney care?” was used throughout to guide the search process and analysis of findings.

## METHODS

### Search strategy

A robust and comprehensive search strategy was employed to identify articles, which addressed or related to barriers and/or facilitators to PS among staff or patients with CKD. A list of search terms was created by reviewing literature surrounding the topic, together with the use of an independent synonym‐generation exercise. A manual search of the reference lists of articles found was also conducted to ensure maximum papers, which answered the research question were included. Robust inclusion/exclusion criteria (Table 1) were developed and applied to ensure that eligibility was consistent. The PICO model was employed throughout the search so the topic could be clearly defined: Problem—CKD or transplant; Intervention—PS; Comparison—barriers/facilitators; Outcome—uptake. No specific publishing timeframe was selected to maximise the number of search results and potential articles to be included. Ethical oversight was not required by any ethics committee for this narrative review, given it only included previously published articles. The PRISMA checklist (Moher, Liberati, Tetzlaff, Altman, & The PRISMA Group, 2009) was used as a reporting guideline to ensure that the narrative review consisted of all necessary components to be considered a sufficient scope of the literature.

**Table 1:** Review search strategy

**Keywords**	**Databases**	**Dates**	**Inclusion criteria**
*P*—Kidney OR Renal OR Dialysis OR Transplant OR Renal replacement therapy OR Chronic kidney disease OR CKD OR End‐stage kidney disease OR End‐stage renal failure	JSTOR	15 October 2019	Studies performed in the United Kingdom
*I*—Peer support OR Peer program* OR Peer service OR Peer group OR Peer advice OR Peer guidance OR Community support OR Community program* OR Community service OR Community group OR Community advice OR Community guidance	EBSCO	18 October 2019	Studies focusing solely or partly on people with kidney disease (CKD or dialysis or transplant)
*C*—Barriers OR Limit OR Challenge OR Obstacle OR Restriction OR Constraint AND Facilitator OR Enabler OR Promoter	Project Muse	20 October 2019	Studies examining participants over 18 years old
*O*—Uptake OR Engage OR Attend OR Experience OR Attitude OR View OR Perception OR Opinion OR Belief OR Behavio*	Medline	21 October 2019	Studies examining peer support programmes for kidney patients individually or in combination with another chronic illness
	CINAHL	22 October 2019	Studies examining one or all of the experiences, barriers and/or facilitators of peer support uptake among kidney patients or staff working on renal units
	Psychinfo		Studies reporting primary research, systematic or literature reviews
	Google Scholar		Studies published in English

Abbreviation: CKD, chronic kidney disease.

### Study selection

Selection of the articles was completed through a two‐step process: Studies in which the title or abstract related to any barriers and/or facilitators to PS among staff or patients with CKD were compiled; subsequently, these studies were then reviewed more in‐depth by two reviewers against the inclusion/exclusion criteria, yielding a finalised list of studies to include in the narrative review. Reasons for exclusion included but were not limited to articles on non‐kidney‐related PS programmes, studies only examining kidney health knowledge or prevalence, and the cost–benefit analysis of PS. Upon collating the findings of the included articles, thematic analysis was applied, which provided insight into general trends throughout the narrative review.

### Thematic analysis

Thematic analysis (Nowell, Norris, White, & Moules, 2017) was employed to identify the most common patterns within the literature. A single reviewer, familiar with the literature, first went through each article and assigned codes accordingly, generating a list. From this list, similar codes were grouped together into categories to create the first set of themes, which were then sent to the other authors to review. Following collaboration and mutual consent between the authors, a finalised list of themes was produced.

## RESULTS

### Search results

**Figure 1:**
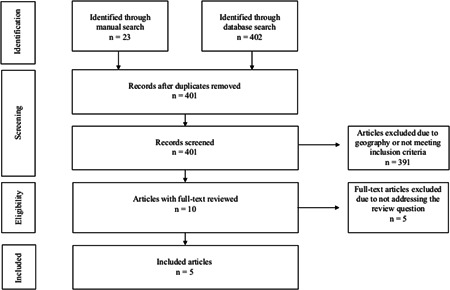
Search selection results

The initial electronic search produced 402 articles in English, reduced to 28 following geographic specifications to the United Kingdom. All 28 titles and abstracts were reviewed against the inclusion/exclusion criteria, and 11 then moved on to the full‐text review stage. Following this, six articles were included as five failed to address the research question. Of the six included, three were deemed “duplicates,” as they discussed the same results as other articles included in the narrative review. Of the manual search of various reference lists, 23 articles were selected to review their abstracts against the inclusion/exclusion criteria. From a more in‐depth cross‐reference, it was found that a number of them were already included in the narrative review, and only an additional two articles were included in the final number. Collectively, the electronic and manual searches yielded five articles to be included in the overall narrative review (Figure 1 and Table 2).

**Table 2:** Articles selected

Author	Title	Methodology	Perspective	Findings
1. Hughes et al. (2009)	Exploring kidney patients' experiences of receiving individual peer support	Qualitative	Patient and staff perspective	Four main themes emerged:
				1. Interaction with the peer supporter 2. Benefits of peer support 3. Contrasts between peer support and other sources of information 4. The peer supporter as a role model
2. Jeffries et al. (2015)	Participation in voluntary and community organisations in the United Kingdom and the influences on the self‐management of long‐term conditions	Qualitative	Patient perspective	1. Reasons for participation (pursuing a hobby to connect to others, participation as a catalyst for change) 2. Barriers to participation (temporal and spatial barriers, group dynamics) 3. Maintaining membership (embedded participation and belonging, involvement and support)
3. McCarthy and Mastin (2016)	Development and delivery of a diverse peer support programme for renal service users, their family and carers: an action research collaboration	Qualitative	Patient and staff perspective	1. Evaluation of peer support training 2. Dissemination 3. Claims, concerns and issues 4. Challenges/barriers 5. Current situation
4. Taylor et al. (2016)	Peer support for CKD patients and carers: overcoming barriers and facilitating access	Qualitative	Patient perspective	1. Perceived benefits of peer support over other sources of support 2. The peer support occasion 3. Permission to engage 4. An attractive peer relationship 5. Building rapport 6. Choice and control
5. Wood (2014)	Patient‐to‐patient peer support in renal care: examining the role and attitudes of renal clinicians	Quantitative and qualitative	Staff perspective	1. Perceived outcomes of peer support 2. Perceptions of the service 3. Suggestions for service improvements

Abbreviation: CKD, chronic kidney disease.

All five articles included mentioned at least one or more of both barriers and facilitators to PS uptake. Four articles assessed barriers and facilitators from a patient perspective (Hughes et al., 2009; Jeffries et al., 2015; McCarthy & Mastin, 2016; Taylor et al., 2016) and three assessed barriers and facilitators from healthcare staff perspectives (Hughes et al., 2009; McCarthy & Mastin, 2016; Wood, 2014). Only two (Hughes et al., 2009; McCarthy & Mastin, 2016) provided insight into both patient and provider perspectives together; however, Wood (2014) also commented on staff's perceived outcomes for PS recipients. Themes surrounding barriers to PS were more common than facilitators among the articles. Following analysis, six main themes became apparent: patient reasons for PS participation; staff barriers to utilising PS; patient barriers to engaging in PS; staff drivers to utilising PS; patient drivers to engaging in PS; positive outcomes of engaging in PS.

Of the number of patient reasons cited for deciding to take part in a PS programme, the most common were informational and emotional support, each listed in three of the five articles, respectively. For informational support, patients chose to participate because they wanted answers to their questions and were seeking information (Hughes et al., 2009; Taylor et al., 2016; Wood, 2014). For emotional support, many patients wanted someone to talk to about what they were going through (Hughes et al., 2009; Jeffries et al., 2015; Wood, 2014). A less significant reason, found in only two articles, was the desire to receive help with treatment decisions (Hughes et al., 2009; Taylor et al., 2016).

### Staff barriers to utilising PS

Low staff referrals were found as the most prominent barrier to people with CKD accessing PS, cited in each of three articles, which assessed staff perspectives (Hughes et al., 2009; McCarthy & Mastin, 2016; Wood, 2014). The most common reasons for low referral rates were that they are considered a time‐consuming and/or difficult process (Hughes et al., 2009; McCarthy & Mastin, 2016; Wood, 2014) and that clinicians only refer a small number of restricted times in the care journey (Hughes et al., 2009; McCarthy & Mastin, 2016; Wood, 2014). Less prominent reasons for low referrals included clinicians being unaware of the referral process (McCarthy & Mastin, 2016; Wood, 2014), concerns the peer supporter could become overburdened (Hughes et al., 2009; Wood, 2014) and the false assumption that nurses, not doctors, are the ones meant to refer (Hughes et al., 2009; McCarthy & Mastin, 2016). A second common staff‐related barrier was difficulty in matching peer supporters to PS recipients (Hughes et al., 2009; McCarthy & Mastin, 2016). While there lacked consensus among the articles reviewed as to a specific reason for this, suggestions included limited availability of a diverse pool of peer supporters (Hughes et al., 2009) and patients having undisclosed preferences for what they are looking for in a peer supporter (McCarthy & Mastin, 2016).

### Patient barriers to engaging in PS

Concern regarding the PS relationship dynamic was found to be the largest deterrent for individuals choosing to engage in PS, found in three articles, which discussed patient‐specific barriers (Hughes et al., 2009; Jeffries et al., 2015; Taylor et al., 2016). Apprehensions were lack of rapport with their peer supporter (Jeffries et al., 2015; Taylor et al., 2016), the peer supporter being negative or frightening (Hughes et al., 2009) and a desire to have a more reciprocal relationship (Taylor et al., 2016). A second barrier, found in two articles, was concern surrounding the format and delivery of PS (Jeffries et al., 2015; Taylor et al., 2016). For instance, lack of time due to other commitments and responsibilities (Jeffries et al., 2015), meeting location challenges (Jeffries et al., 2015) and a desire to choose the timing and delivery of the support (Taylor et al., 2016) were some specific examples of format and delivery barriers.

### Staff drivers to utilising PS

Promoting PS among clinicians was found to be the only significant facilitator enabling staff to utilise PS, cited in each of the three articles on staff perspectives (Hughes et al., 2009; McCarthy & Mastin, 2016; Wood, 2014). A successful method of promotion with clinicians was having link nurses raise awareness about PS and its associated benefits through announcements at meetings, emails and informal conversations with their colleagues (Hughes et al., 2009; McCarthy & Mastin, 2016; Wood, 2014). Another suggestion was to have leaflets in consulting rooms in outpatient clinics as a mode of positive reinforcement for clinicians to remember to refer (Hughes et al., 2009).

### Patient drivers to engaging in PS

An inclusive service was the leading factor for people choosing to engage in PS, referenced in three articles assessing patient drivers (Hughes et al., 2009; Jeffries et al., 2015; Taylor et al., 2016). Although there lacked a common definition of what an inclusive service entails, suggestions included allowing self‐referrals (Hughes et al., 2009), having recipients play a key role in choosing their peer supporter (Taylor et al., 2016) and ensuring the support is accommodating and catered to the patient (Jeffries et al., 2015).

### Positive outcomes of engaging in PS

The most frequent positive outcome of engaging in PS, listed in all five articles, was the interaction providing an opportunity for the patient to receive emotional support (Hughes et al., 2009; Jeffries et al., 2015; McCarthy & Mastin, 2016; Taylor et al., 2016; Wood, 2014). Secondly, PS was also established as a helpful approach for recipients to gain information and answers to their questions (Hughes et al., 2009; Jeffries et al., 2015; Taylor et al., 2016; Wood, 2014). Lastly, PS was cited as a positive experience because it provides reassurance for people and helps them increase their confidence in decision‐making (Hughes et al., 2009; Jeffries et al., 2015; Taylor et al., 2016).

## DISCUSSION

The narrative review's findings have identified staff‐ and patient‐related barriers and facilitators to PS uptake among people with CKD in the United Kingdom and poses useful suggestions for how to address them accordingly. This discussion aims to analyse such findings, identify areas for improvement and suggest recommendations for practice.

### Overcoming healthcare staff barriers

Healthcare staffs have a significant impact on a patient's level of engagement, and a programme's success is directly correlated to the amount of support and referrals made by clinicians (McCarthy & Mastin, 2016). PS referral processes have been found to be considered mysterious, time‐consuming and/or difficult and some clinicians only consider referring at specific times in the care journey. Moreover, staffs have stated it can be challenging to match recipients with peer supporters based on similarities if there is a limited peer supporter pool and if recipients have preferential traits they are looking for. Although matching requires a time commitment and the requisite knowledge of who is available, pairing recipients with peer supporters as closely as possible makes the service immensely more successful and effective (Hughes et al., 2009).

By promoting PS among clinicians, specifically through increased training and awareness on service practicalities, governance, and PS benefits, staff may, in turn, increase their referrals. Greater exposure nationally and internationally for PS may also improve clinician confidence in the service and aid as a reminder to refer throughout the entire care journey. Since some clinicians may unintentionally recommend PS more to some patient groups over others, the effort is therefore needed to avoid these assumptions to promote equality of access (Krizek, Roberts, Ragan, Ferrara, & Lord, 1999). For instance, PS opportunities offered through national organisations could help to increase knowledge and understanding of the benefits (McCarthy & Mastin, 2016). Notably, the promotion of PS among clinicians was the main staff facilitator found in the narrative review and can have a significant impact (Wood, 2015). By increasing awareness, more staff will also be aware of who could be recruited as a peer supporter, which would aid in increasing diversity and expanding the number of peer supporters. Crucially, ensuring clinicians understand how their engagement affects the overall success of PS could enhance their motivation to refer as well as support with the recruitment and matching process. For instance, past potential recipients have stated their desire for clinicians to affirm that PS is appropriate for them and that they may not engage unless they receive such confirmation (Taylor et al., 2016). Ultimately, if staff are confident and motivated to frequently promote PS to patients, the opportunities for people to engage will be maximised (Wood, 2014).

### Overcoming patient barriers

Having peer supporters who are welcoming and comfortable to engage with is seen as imperative among a number of recipients. The promotion and explanation of PS to potential recipients, which emphasises that supporters are trained and expected to be positive and understanding role models may alleviate potential fears (Hughes et al., 2009). As found by Taylor et al. (2016), the term “peer support” can be perceived negatively or entirely misunderstood, so framing it in a way, which highlights its informality and clarifying the true intention and potential positive outcomes may attenuate concerns. An additional opportunity for PS recipients to provide feedback to clinicians about peer supporters could also reassure recipients that they will have support even if there are issues with the relationship dynamic. Ensuring supporters are taught during training sessions about the significance of providing a safe and empathetic environment could also have a sizeable impact on tackling this barrier.

Ultimately, the flexibility of the programme's format and delivery is essential to ensure ample opportunity for access (Taylor et al., 2016). This highlights what was found as a prominent facilitator in the narrative review, providing an inclusive PS service. The timing of PS can influence whether a person will participate or not, given that individuals desire PS at different times throughout their disease trajectory (Embuldeniya et al., 2013; Smith et al., 2011) and reach acceptance of external support at varying times (Taylor et al., 2016). Thus, there is not a single time point at which individuals should be targeted for involvement in PS, and instead, they should be given as many options as possible of when and how to access the service (McCarthy & Mastin, 2016; Wood, 2014). This may mean having the opportunity to self‐refer or to have more control during the selection process of their peer supporter. Moreover, concerns such as travel arrangements, lack of available time, or fear of initial meetings could also be alleviated with a flexible format, such as options to speak over the phone, internet, or in‐person and for the PS recipient to influence/choose the date/time of the session (Wood, 2014). Liaghat (2017) found in‐person sessions, particularly effective, while Nicholas et al. (2009) stated that online delivery showed promise; having greater selection gives recipients more options to choose from.

### Motivations for patients

Of the reasons for people choosing to get involved in PS, informational, emotional and decision‐making support, all were identified as significant. An opportunity to learn more about their condition and have support in making decisions surrounding their care has also been found as common motivators in studies assessing PS in cancer (Lu, You, Man, Loh, & Young, 2014) and cardiovascular disease (Wright et al., 2001). To ensure individuals are aware that PS is an opportunity to gain access to information and help with decision‐making, accurate promotion of PS by clinicians is crucial (Wood, 2014). Additionally, framing PS in a way that identifies peer supporters as role models may help in normalising how patients feel and give them an incentive to get involved (Hughes et al., 2009).

### Implications for clinical practice and research

National policy recommends that every person in the United Kingdom with CKD have access to PS; however, this narrative review highlights that there are a number of barriers, which impede the uptake of PS. As there are few studies that assess PS in kidney care, there is plenty more to be learned at a national and international level about what both patients and clinicians have experienced with PS to enable more people to benefit.

Existing evidence suggests numerous strategies units can employ if they struggle with overcoming staff‐ or patient‐related barriers. First, clinicians need the training to understand the likely benefit of PS and the logistics of the local PS service, so that they feel confident and motivated to discuss the service with and refer potential recipients. Research evidencing the most effective methods of increasing clinician awareness would be valuable. Second, PS services need to be designed to be as inclusive as possible to allow recipients to assess PS at the time and in a way that suits them personally. This means offering PS in a variety of formats and at all stages of kidney disease, and so on. It is recommended to have individuals passionate about PS take the lead with its organisation, and to consult patients and carers, so the service is tailored to the needs of people with kidney disease (Wood, 2014). Furthermore, it will be essential to have investment from senior level management and a consistent set of volunteers to help sustain the programme.

As only two articles in the narrative review assessed both patient and healthcare professional perspectives simultaneously, there is a need for further studies to evaluate both. Moreover, many of the articles focused solely on one unit within the UK, so more studies need to be carried out on a national and international level to ensure the results are more generalisable to the greater population. While there have been studies within this context in other countries such as the United States (Liaghat, 2017) and Canada (Nicholas et al., 2009), more work within this field could increase the overall awareness and prioritisation of PS in kidney care.

### Limitations

Given the nature of a narrative review, several limitations must be mentioned when assessing its value. First, due to the small number of articles included, the findings should not be considered an entire comprehensive overview of all barriers and facilitators to PS in kidney care. Although a rigorous and specific search strategy was employed, there may also have been some subjectivity in the actual selection of which articles were included, as well as during the thematic analysis. Moreover, given many of the articles' qualitative methodology, the results cannot be considered generalisable to the UK population. Despite such limitations, the findings still present a thorough overview and understanding of the topic and offer valuable suggestions for future practice and research.

## CONCLUSION

This narrative review has highlighted the most prominent staff‐ and patient‐related barriers and facilitators for the uptake of PS among people with CKD in the United Kingdom within the current literature. Among the staff barriers, low referrals and difficulty matching peer supporters deterred PS from being successful, with specific reasons behind each barrier identified. For staff‐related facilitators, promoting PS with clinicians aided its overall success. The main patient barriers cited were concerns regarding the peer supporter relationship dynamic and the actual format and delivery of the service. Having an inclusive service, which provided the recipient with greater autonomy in decision‐making, was the primary facilitator among patients. Recommendations for clinical practice include promoting PS with clinicians to increase their motivation to refer and empowering people to access PS, which suits their specific needs. As a number of studies did not review patient and provider perspectives collectively, further study is required to gain a more holistic understanding of the barriers and facilitators to the uptake of PS. Future studies should include kidney units throughout the United Kingdom and internationally that already have PS established to understand what has helped or impeded its success, as well as units that have yet to implement a PS service to learn what would be required for initiation.

## CONFLICT OF INTERESTS

The authors declare that there are no conflict of interests.

## AUTHOR CONTRIBUTIONS

Andrew Trasolini is responsible for designing and carrying out the search strategy, article selection, thematic analysis and writing of the narrative review. He is responsible for submitting to the *Journal of Renal Care*. Eleri Wood is responsible for article selection and contributing to the writing and editing of the narrative review. She read and reviewed the final draft. Nicola Thomas is responsible for contributing to the writing and editing of the narrative review. She read and reviewed the final draft.
